# Comparison of hospitalizations in patients on first generation versus second generation long acting injectable (LAI) antipsychotics

**DOI:** 10.1192/bjo.2021.753

**Published:** 2021-06-18

**Authors:** Seemab Rasool, Paster Venan

**Affiliations:** Essex Partnership University NHS Foundation Trust

## Abstract

**Aims:**

There is limited data on the comparison of efficacy between first and second antipsychotic LAIs. One good indicator of efficacy is the rates of hospitalization. Some studies have shown that second generation depot antipsychotics, significantly reduce hospitalizations as compared to conventional depots.

Our aim was to compare hospitalizations in patients on first and second generation LAI antipsychotics.

**Method:**

A retrospective observational study was done by reviewing the records of all the depot clinics in South Essex, United Kingdom. A list of patients enrolled and receiving LAI antipsychotics was obtained from the 6 depot clinics. Data were collected by going through the electronic records of the patients on the depot clinic lists and taking down the demographics, diagnosis and the hospital admissions. Other variables like comorbid drug abuse were also recorded.

**Result:**

Amongst a total of 346 patients 223 (64 %) were males and 123 (36%) were females. Average age was 50.3 (range 21 to 88 years) and 290 (83%) patients were single. An overwhelming majority of patients 299 (87 %) were not in employment. Regarding the diagnosis, the majority, 237 patients were diagnosed with Paranoid Schizophrenia, 49 patients were diagnosed with Schizoaffective disorder, 38 patients were diagnosed with Bipolar affective disorder, 20 patients had a diagnosis of Delusional disorder and only 2 patients had a primary diagnosis of Mental and Behavioral disorders due to substance abuse. Of the total 346 only 17 patients were on a Community treatment Order.

Risperidone was the most commonly used second generation LAI at 26%,Aripiprazole in 10% and Paliperidone was used in 5% patients. Olanzapine LAI was only used in 2 patients. Amongst first generation LAIs Zuclopenthixol, Fluclopentixol were both used in 24%, and Haloperidol in 10% patients. 21 % of patients were reported to be actively abusing drugs.

65 (32.6%) of the total 200 patients on Ist Generation LAIs had hospital admissions

55 (39.8%) of the total 138 patients on 2nd Generation LAIs had hospital admissions

This difference was not statistically significant (Z test)- P value of 0.082427

**Conclusion:**

The results in our observational study are equivocal, both LAIs providing equitable decrease in the hospital admissions albeit with a slightly favourable outcome (not statistically significant though) attributable to the first generation LAIs. There was a high incidence of unemployment and drug abuse in our cohort of patients, thus targeted interventions can be established in rehabilitation of such individuals.

